# High-Order Quantum-Mechanical Analysis of Hydrogen Bonding in Hachimoji and Natural DNA Base Pairs

**DOI:** 10.1021/acs.jcim.3c00428

**Published:** 2023-05-01

**Authors:** Rameshwar L. Kumawat, C. David Sherrill

**Affiliations:** Center for Computational Molecular Science and Technology, School of Chemistry and Biochemistry, School of Computational Science and Engineering, Georgia Institute of Technology, Atlanta, Georgia 30332-0400, United States

## Abstract

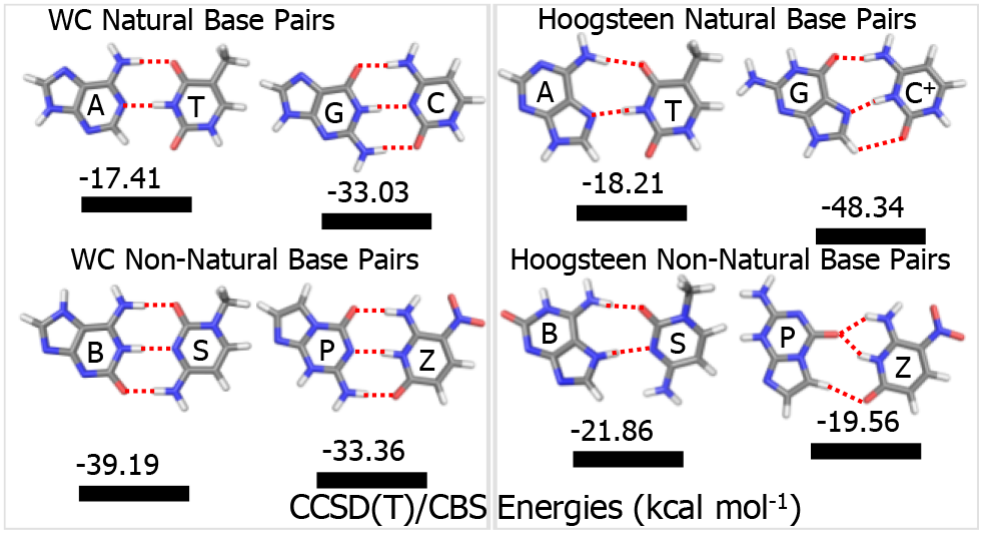

High-order quantum chemistry is applied to hydrogen-bonded natural DNA nucleobase pairs [adenine:thymine (A:T) and guanine:cytosine (G:C)] and non-natural Hachimoji nucleobase pairs [isoguanine:1-methylcytosine (B:S) and 2-aminoimidazo[1,2a][1,3,5]triazin-4(1H)-one:6-amino-5-nitropyridin-2-one (P:Z)] to see how the intermolecular interaction energies and their energetic components (electrostatics, exchange-repulsion, induction/polarization, and London dispersion interactions) vary among the base pairs. We examined the Hoogsteen (HG) geometries in addition to the traditional Watson–Crick (WC) geometries. Coupled-cluster theory through perturbative triples [CCSD(T)] extrapolated to the complete basis set (CBS) limit and high-order symmetry-adapted perturbation theory (SAPT) at the SAPT2+(3)(CCD)δMP2/aug-cc-pVTZ level are used to estimate highly accurate noncovalent interaction energies. Electrostatic interactions are the most attractive component of the interaction energies, but the sum of induction/polarization and London dispersion is nearly as large, for all base pairs and geometries considered. Interestingly, the non-natural Hachimoji base pairs interact more strongly than the corresponding natural base pairs, by −21.8 (B:S) and −0.3 (P:Z) kcal mol^–1^ in the WC geometries, according to CCSD(T)/CBS. This is consistent with the H-bond distances being generally shorter in the non-natural base pairs. The natural base pairs are energetically more stabilized in their Hoogsteen geometries than in their WC geometries. The Hoogsteen geometry makes the A:T base pair slightly more stable, by −0.8 kcal mol^–1^, and it greatly stabilizes the G:C^+^ base pair, by −15.3 kcal mol^–1^. The G:C^+^ stabilization is mainly due to the fact that C has typically added a proton when found in Hoogsteen geometries. By contrast, Hoogsteen geometries are substantially less favorable than WC geometries for non-natural Hachimoji base pairs, by 17.3 (B:S) and 13.8 (P:Z) kcal mol^–1^.

## Introduction

Noncovalent interactions (NCI) play a key role in many applications in (bio)chemistry/physics, materials and nanoscience, and biology. NCI govern such processes as drug binding, supermolecular assembly, and crystal packing of organics. In biochemistry, the architectures of biomacromolecules such as deoxyribonucleic acid (DNA), ribonucleic acid (RNA), and proteins are determined by NCI among the building blocks.^1,2^

In 1953, James Watson and Francis Crick reported a double helix structure for double-stranded DNA.^3^ This double helix structure assembles through H-bonding between the nucleobases to form Watson–Crick (WC) base pairs: adenine (A) with thymine (T), and guanine (G) with cytosine (C). For RNA, T is replaced with uracil (U). A decade after Watson and Crick published their model of the DNA double helix, Karst Hoogesteen discovered that A:T(U) and G:C^+^ can pair in a different geometrical orientation now called Hoogsteen pairing (here C^+^ denotes a protonated cytosine).^4^

In 2014, Malyshev and co-workers opened up a novel technique in artificial (or synthetic) biology by introducing exogenous non-natural nucleic acid base pairs into a living organisms DNA, demonstrating the feasibility of propagating an augmented genetic alphabet.^5^ Further advances in synthetic biology have demonstrated the creation of semisynthetic DNA- and RNA-like systems built from eight nucleotide “letters,” the original four for DNA (or RNA), plus four non-natural so-called “Hachimoji” (HM) nucleobases, allowing a total of four orthogonal pairs in DNA or RNA.^6^ Analogous to natural double-stranded DNA and RNA, these non-natural nucleobases can also form double helices. Along with natural nucleobase H-bond pairs, Hachimoji DNA and RNA can involve additional H-bond pairs resulting from new synthetic nucleobases: isoguanine (B), 2-aminoimidazo[1,2a][1,3,5]triazin-4(1H)-one (P), isocytosine (rS), 1-methylcytosine (S), and 6-amino-5-nitropyridin-2-one (Z).^6^ These are the Hachimoji nucleobases synthesized by Hoshika and co-workers in early 2019.^6^ The non-natural DNA nucleobase S bonds with B, and P bonds with Z, while B bonds with rS in the non-natural RNA system. These non-natural DNA building blocks P and B are analogues of the *purine* system, and S (and rS) and Z are analogues of the *pyrimidine* system. These synthetic systems meet the structural requirements needed to support Darwinian evolution, including a polyelectrolyte backbone, predictable thermodynamic stability, and stereoregular building blocks that fit a Schrödinger aperiodic crystal.^6^ Like natural DNA, Hachimoji DNA though non-natural can also support the evolution of organisms.^4^ Non-natural DNA has been suggested for numerous applications, including information storage and drug design.^6−9^

It is well established that natural DNA and RNA duplexes are primarily stabilized by the forces of WC H-bonding and nucleobase stacking (interstrand and intrastrand).^10−12^ The H-bonds are stronger than the π-stacking interactions, although both are essential for the stability of natural DNA and RNA duplexes in aqueous solution.^12−14^ It has been reported that H-bonding is primarily electrostatic in nature with a non-negligible dispersion contribution.^15,16^ Jurečka and co-workers have reported^16−18^ high-level computations for H-bonding interaction energies of several DNA base pairs, including some with nonstandard substituents, using second-order Møller–Plesset perturbation theory (MP2) with coupled-cluster corrections including noniterative triples contributions [CCSD(T)].^19^ Hesselmann and co-workers have examined^20^ the interaction energy between A:T and G:C base pairs and their physical components using density functional theory-based symmetry-adapted perturbation theory (DFT-SAPT),^21,22^ and Fonseca Guerra and Bickelhaupt conducted a similar study^23^ using an energy decomposition scheme of the supermolecular DFT energy.

However, to date, no high-level theoretical study has directly compared H-bonding in non-natural Hachimoji nucleobases to that in natural WC base pairs. Here, we present such an analysis using high orders of symmetry-adapted perturbation theory (SAPT) to obtain the electrostatic, exchange-repulsion, induction/polarization, and London dispersion contributions to the interaction in each base pair, complemented by CCSD(T) computations in the complete basis set (CBS) limit to obtain benchmark-quality interaction energies. Our analysis includes Watson–Crick-type geometries, as well as Hoogsteen-type geometries (newly proposed here for the Hachimoji base pairs). We analyze interaction energies and their components in terms of the geometries of the base pairs and find in general that interaction strength in these systems correlates with the sum of H-bond and H-bond-like closest-contact distances.

## Theoretical Methods

In this present study, we have modeled a total of eight base pairs: natural WC base pairs (A:T and G:C) and non-natural HM base pairs (P:Z and B:S), as well as Hoogsteen (HG) geometry base pairs (A:T and G:C^+^) and HG-type HM base pairs (P:Z and B:S). We employ B3LYP-D3(BJ)^24−26^ methods that have become routinely applied to NCI geometry optimizations, with the popular correlation-consistent basis sets of Dunning augmented with diffuse functions [aug-cc-pVXZ (X = D, T, Q); abbreviated throughout as aXZ].^27^ All three basis sets provide essentially identical geometries. The geometry optimization was done using the Q-Chem code.^28^ Geometries were also optimized using (frozen core) MP2, and results were very similar to the DFT geometries. We used the B3LYP-D3(BJ)/aDZ geometries for further analysis. All results are obtained in the gas phase, which is sufficient to quantify the intrinsic attraction between the nucleobases. In the condensed phase, additional interactions will be present (e.g., interactions between the nucleobases and water molecules and also nearby nucelobases and DNA backbone), but the details will vary depending on the particular environment. Moreover, such additional interactions are not expected to modify or “tune” the direct nucleobase–nucleobase interactions.^29^

At the DFT geometries, we obtained high-quality intermolecular interaction energies. To obtain noncovalent interaction energies as well as their physically meaningful components, we used the “gold standard”^30^ of symmetry-adapted perturbation theory, i.e., SAPT2+(3)(CCD)δMP2/aXZ, where X = D, T. This level of theory includes the following terms:
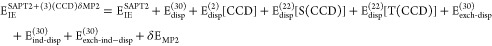
1where
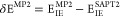
2and
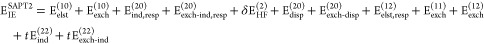
3

IE denotes an interaction energy. The two numbers in superscripts refer to the order of the perturbation for the intermolecular interaction and the intramolecular electron correlation, respectively. Cross terms like exchange-induction and exchange-dispersion are accounted as induction and dispersion, respectively. A detailed discussion of the above terms can be found in our previous paper.^30^ This level of SAPT is expected to provide accurate noncovalent interaction energies and their components. Interaction energies should similar to those from the reliable CCSD(T)/CBS method.^30^ We also considered some lower levels of SAPT for comparison purposes.

Our most accurate estimates of the interaction energies are obtained using the focal-point approach to estimate the CCSD(T)/CBS limit,^31,32^ whereby the MP2/CBS limit [computed using the two-point extrapolation scheme of Helgaker,^33^ denoted as MP2/CBS(aXZ, a[X+1]Z)] is corrected for higher-order electron correlation effects by adding the difference between CCSD(T) and MP2 as computed in a smaller basis set, denoted by δ_MP2_^CCSD(T)^. In particular, these noncovalent interaction energy values are computed at the MP2/CBS(aTZ, aQZ) + δ_MP2_^CCSD(T)^/X and MP2/CBS(aQZ, a5Z) + δ_MP2_^CCSD(T)^/X levels of theory, where X = aDZ or aTZ. These computations will be abbreviated here as CCSD(T)/CBS[aTQZ;δ:X] and CCSD(T)/CBS[aQ5Z;δ:X]. This focal-point approach has been widely used to estimate CCSD(T) at the CBS limit for noncovalent interaction energy values.^18,34−49^

CCSD(T) interaction energies are computed with and without the Boys–Bernardi counterpoise correction.^50−52^ Core electrons are kept frozen in all interaction energy computations, which have been performed with the quantum chemistry program Psi4 v1.5.^53,54^

## Results and Discussion

### Watson–Crick Natural and Hachimoji Non-Natural DNA Base Pairs

**Figure 1:**
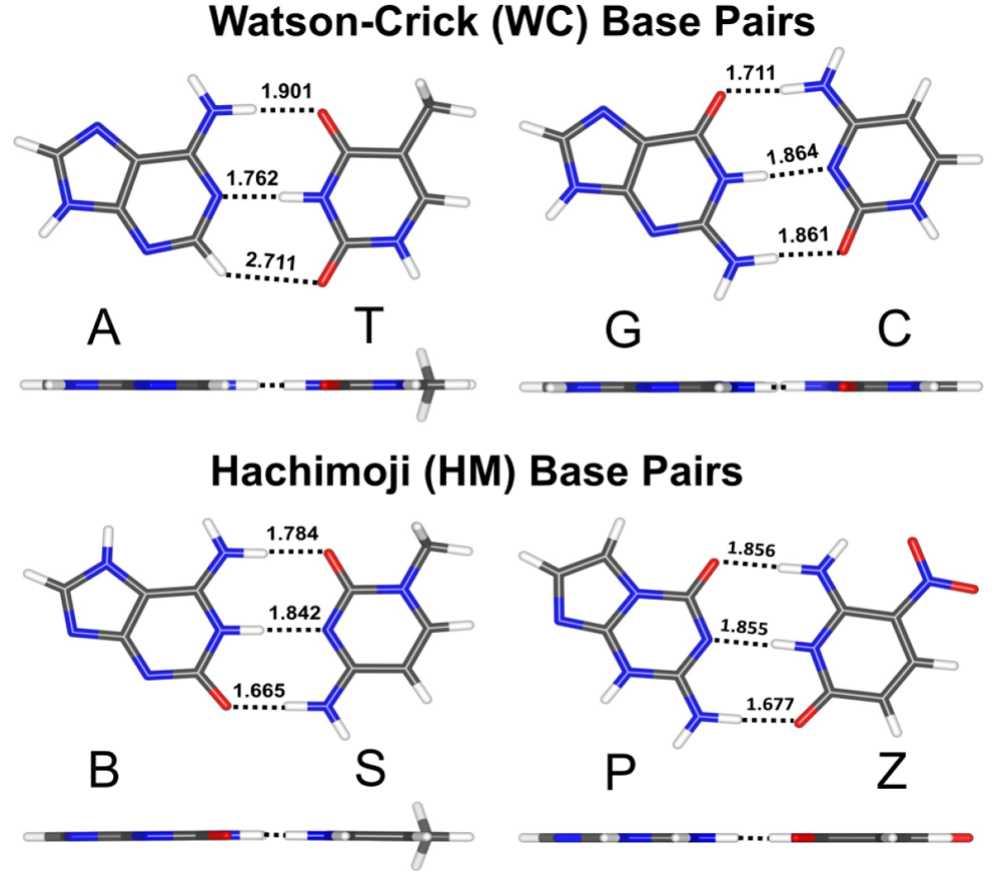
Optimized structures of the H-bonded natural WC (A:T, G:C) and non-natural HM (B:S, P:Z) base pairs at the B3LYP-D3(BJ)/aug-cc-pVDZ basis set level of theory. The dotted black color lines represent the H-bond distances (Å) for different nucleic acid base pairs.

Optimized structures of the four H-bonded nucleic acid base pairs are shown in Figure 1. The DFT computed structures show planar H-bonded structures for the natural base pairs (A:T and G:C) and non-natural base pairs (B:S and P:Z). The computed H-bonding closest-contact distances (distance between hydrogen being donated and the acceptor atom, in Å) are included in the figure. Each base pair features three H-bonds with intermolecular contact distances about 1.9 Å or less, except for A:T which has two such bonds plus a long 2.7 Å weak C–H···O bond.

High-quality total interaction energy values approaching the CCSD(T)/CBS limit, along with our highest level SAPT computations at the SAPT2+(3)(CCD)δMP2/aTZ level of theory, are presented in Table 1 for the WC-type (A:T, G:C, B:S, P:Z) and HG-type (A:T, G:C^+^, B:S, P:Z) nucleic acid base pairs. Table S1 of the Supporting Information additionally presents CCSD(T)/CBS results without counterpoise correction, and also using the smaller aDZ basis set for the CCSD(T) correction, to demonstrate basis set convergence. The difference in interaction energies between the CCSD(T)/CBS[aTQZ;δ:aTZ] and CCSD(T)/CBS[aQ5Z;δ:aTZ] computations is very tiny and amounts to ≤0.02 kcal mol^–1^ for the WC- and HG-type base pairs, indicating that the MP2 portion of the interaction energy is very well converged. The differences between CCSD(T)/CBS[aQ5Z;δ:aTZ] and CCSD(T)/CBS[aQ5Z;δ:aDZ] (provided in the SI) are rather small (ranging from 0.2 to 0.4 kcal mol^–1^ with counterpoise correction, and 0.0 to 0.1 kcal mol^–1^ without counterpoise correction) and suggest that aTZ is probably sufficient to converge the coupled-cluster part of the focal point procedure to within ∼0.1–0.2 kcal mol^–1^. Additionally, the interaction energy difference between counterpoise correction and without counterpoise correction ranges from 0.1 to 0.2 kcal mol^–1^ for CCSD(T)/CBS[aQ5Z;δ:aDZ] and 0.0 to 0.1 kcal mol^–1^ for CCSD(T)/CBS[aQ5Z;δ:aTZ] for WC-type base pairs. This suggests that any remaining basis set superposition error in our best estimates is rather small. We may also compare our best total interaction energy values at the CCSD(T)/CBS limit to the CCSD(T)/CBS[aTQZ;δ:DZ] results reported by Jurečka and co-workers^18^ for the A:T and G:C base pairs. Despite using a methodology that appears similar, by neglecting diffuse functions for the δ_MP2_^CCSD(T)^ correction, the interaction energies of Jurečka et al. are underbinding by 0.5–0.9 kcal mol^–1^ vs our best estimates with larger basis sets.

**Table 1:** Total Interaction Energy (in kcal mol^–1^) of WC-Type and HG-Type Nucleic Acid Base Pairs

Base Pairs	WC type				HG type			
Methods	A:T	G:C	B:S	P:Z	A:T	G:C^+^	B:S	P:Z
CCSD(T)/CBS[aQ5Z;δ:aTZ]	–17.41	–33.03	–39.19	–33.36	–18.21	–48.34	–21.86	–19.56
CCSD(T)/CBS[aTQZ;δ:aTZ]	–17.40	–33.01	–39.17	–33.34	–18.20	–48.32	–21.85	–19.55
SAPT2+(3)(CCD)δMP2/aTZ	–16.87	–32.82	–39.30	–33.11	–17.63	–48.22	–22.00	–19.59
CCSD(T)/CBS[aTQZ;δ:DZ]^a^	–16.9	–32.1						
DFT-SAPT/aTZ^b^	–15.2	–29.8						

a Taken from Jurečka et al.^18^ The authors used modified 6-31G** and cc-pVDZ basis sets for the δ_MP2_^CCSD(*T*)^ correction, and they do not specify which one is used for A:T and G:C computations.

b Taken from Hesselmann et al.^20^

Results from our highest-level SAPT method, SAPT2+(3)(CCD)δMP2/aTZ, are quite close to our best CCSD(T)/CBS estimates. The range of differences between SAPT2+(3)(CCD)δMP2/aTZ and CCSD(T)/CBS[aQ5Z;δ:aTZ] is from −0.11 to 0.54 kcal mol^–1^ for WC-type natural and non-natural base pairs. This indicates that our highest level of SAPT should be quite suitable for analyzing the various components of the interaction energy; we present this analysis below. In Tables S3–S4 and Figures S4–S11, we present a comparison between different levels of SAPT with aTZ and aDZ basis sets. Changes of a few kcal mol^–1^ in the interaction energies are found as we add more terms in SAPT. The modest changes in interaction energy when the basis set increases from aDZ to aTZ suggest that the SAPT results using the aTZ basis are fairly well converged with respect to the basis set. Finally, Table 1 also provides a comparison to the DFT-SAPT/aTZ results of Hesselmann et al.^20^ for the A:T and G:C base pairs. Their results are underbound by 2.2 (A:T) and 3.2 (G:C) kcal mol^–1^ compared to our highest-level CCSD(T)/CBS results, for errors of about 10%–13%. SAPT2+(3)(CCD)δMP2/aTZ reduces the underbinding to 0.5 and 0.2 kcal mol^–1^, respectively (or about 1%–3%), albeit at a significantly increased computational cost compared to DFT-SAPT.

The natural G:C base pair has nearly twice the interaction energy as the natural A:T base pair (Table 1). These interaction energies are determined primarily by the H-bonds formed between base pairs. We note that the G:C base pair has 3 H bonds, and A:T has only 2 H-bonds and a long weak C–H···O bond (Figure 1). For the non-natural base pairs, the B:S base pair has a somewhat more negative interaction energy than the P:Z base pair (by 5.8 kcal mol^–1^). Two of the bond lengths are very similar for B:S and P:Z, but one is somewhat weaker (lengthened by 0.072 Å) in the P:Z base pair (Figure 1). Comparing the H-bond lengths in G:C vs P:Z, which have very similar interaction energies, we see that the middle H-bond is about the same length in both complexes (shorter by 0.009 Å in P:Z), while the top H-bond in the figure lengthens by 0.145 Å in P:Z, and the bottom H-bond shortens by 0.184 Å. These bond length changes thus seem to approximately cancel out in the determination of the overall interaction energies of G:C vs P:Z. Indeed, the sum of the three closest-contact H-bond distances in each base pair (including the weak C–H···O bond in A:T) seems to correlate well with the total interaction energy: 6.374 Å and −17.4 kcal mol^–1^ (A:T), 5.436 Å and −33.0 kcal mol^–1^ (G:C), 5.388 Å and −33.4 kcal mol^–1^ (P:Z), and 5.291 Å and −39.2 kcal mol^–1^ (B:S).

#### SAPT Energy Component Analysis

**Figure 2:**
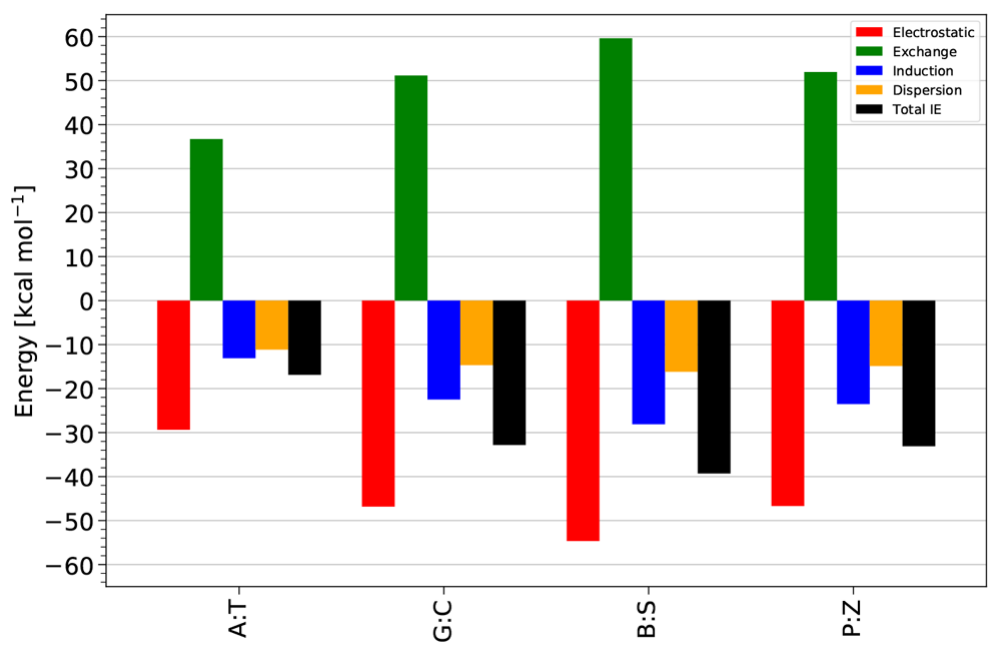
Total noncovalent interaction energy (total IE) values and SAPT energy components (electrostatic, exchange, induction, dispersion) for the WC (A:T, G:C) and HM (B:S, P:Z) nucleic acid base pairs at the SAPT2+(3)(CCD)δMP2/aTZ basis set level of theory.

The point of view that H-bonding is mainly stabilized by electrostatic contributions^20^ is supported by Figure 2, in the sense that it is the dominating attractive contribution. In addition, the electrostatic term varies the most between base pairs. However, note that the attractive electrostatic contributions are not enough to overcome the exchange-repulsion terms. Indeed, the sum of electrostatics and exchange-repulsion is +4.3–7.4 kcal mol^–1^ for the A:T, G:C, B:S, and P:Z base pairs, respectively (detailed energy component data are provided in the Supporting Information Tables S5 and S6). Figure 2 shows that induction and London dispersion components are also contributing significantly to total interaction energy values. While for the A:T base pair, the induction and dispersion energy contributions are nearly similar (within 2.0 kcal mol^–1^); for other base pairs, induction is significantly larger than dispersion, by 7.8 kcal mol^–1^ or more. Overall, all four components are very important for determining the interaction energy. For the B:S base pair, the electrostatic contribution is the strongest among the base pairs. Furthermore, we note that the electrostatic, exchange-repulsion, induction, and London dispersion components for the B:S base pair are stronger by at least −8.0, 7.7, – 4.6, and −1.3 kcal mol^–1^ than in the A:T, G:C, and P:Z base pairs. SAPT interaction energy components tend to be stronger for intermolecular interactions with shorter contacts, and as noted above, the sum of the H-bond contact distances is the shortest (5.291 Å) for B:S. P:Z and G:C have energy components that are similar to each other, but smaller in magnitude than for B:S. This is consistent with the sum of H-bond contact distances being larger for these dimers than for B:S, but similar to each other (5.388 Å for P:Z and 5.436 Å for G:C). Finally, the SAPT energy components are the smallest in magnitude for A:T, which has only two real H-bonds plus a weak C–H···O contact (summing to 6.374 Å).

### Hoogsteen Natural and Hoogsteen-Type Non-Natural Hachimoji DNA Base Pairs

**Figure 3:**
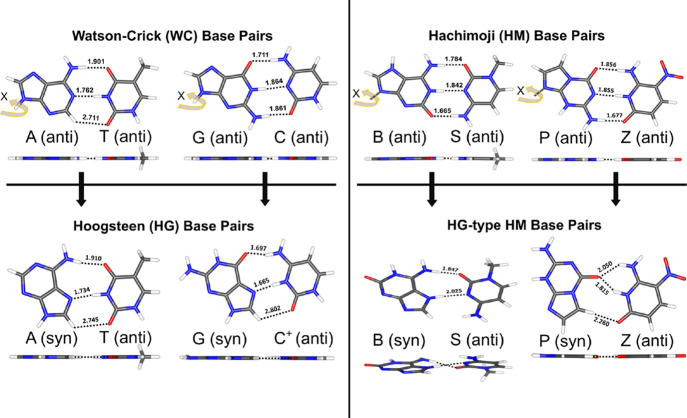
Optimized structures of the HG (A:T, G:C^+^) and HG-type HM (B:S, P:Z) nucleic acid base pairs at the B3LYP-D3(BJ)/aDZ basis set level of theory. The dotted black color lines represent the H-bond distances (Å) for different nucleic acid base pairs.

Next, we consider natural Hoogsteen (HG) base pair geometries, to form A(syn):T(anti) and G(syn):C^+^(anti), by rotating the purine base in the WC-type base pair 180° around the glycosidic bond torsion angle, so that it adopts a syn conformation (0° < χ < 90°, where χ is the glycosidic bond torsion angle as illustrated in Figure 3 and Figure S1) rather than an anticonformation (−180° < χ < −90°), followed by the translation of the two bases by ∼2.0–2.5 Å, thus allowing the formation of a set of H-bonds (Figure 3 and Figure S1).^55−58^ In a similar way, we create HG-type HM base pairs to form B(syn):S(anti) and P(syn):Z(anti). Optimized structures of natural HG base pairs and a non-natural P:Z HG geometry are found to be planar, while the non-natural B:S HG geometry turns nonplanar. We suspect that unfavorable H···H intermolecular interactions [H from B (syn) and NH_2_ from S(anti), Figure 3] might be responsible for the nonplanar geometry of the HG B:S base pair.

The interaction energy results at the equilibrium geometries of the HG-type base pairs are tabulated in Table 1 and Table S1. Additional comparison tables of interaction energy results for CCSD(T)/CBS with and without counterpoise correction and with different SAPT approaches and basis sets are also presented in the Supporting Information. As indicated in Table 1 and Tables S1 and S2, the interaction energies of the HG-type base pairs are in excellent agreement with each other as we vary the basis sets used in the CCSD(T)/CBS estimates, or when we compare those values to our highest-level SAPT values (within a few tenths of one kcal mol^–1^). This is consistent with our findings for the WC geometries. Herein, we mainly discuss the results obtained using the highest-level CCSD(T)/CBS[aQ5Z;δ:aTZ] focal-point approach and the SAPT2+(3)(CCD)δMP2 approach.

From Table 1, we note that the HG-type (A:T, G:C^+^) base pairs are more stabilized (have more negative interaction energies) than the WC-type base pairs. The interaction energy differences at our best level of theory, CCSD(T)/CBS[aQ5Z;δ:aTZ], are −0.8 kcal mol^–1^ for A:T and −15.3 kcal mol^–1^ for G:C^+^. For the Hoogsteen G:C^+^ base pair, the N–H···O contact distance is slightly shorter, and the N–H···N distance is significantly shorter than in the WC geometry. However, the final N–H··· O H-bond is replaced with a weak C–H···O contact (2.802 Å), which one might expect to lead to a much weaker overall interaction energy in the HG geometry. Instead, the interaction is much stronger in the HG geometry because of the protonation of C, leading to strong ion–dipole interactions. This explanation is confirmed by the SAPT analysis below.

The H-bond contact distances for A:T are similar in the HG geometry as in the WC geometry, consistent with its similar binding energy. The slightly stronger interaction of the A:T base pair in the HG geometry than in the WC geometry is consistent with the experimental infrared-spectra analysis of the A:T base pair in a nonpolar solvent.^59−61^ This result of a stronger interaction in the HG geometry is also consistent with previous quantum chemical studies by Hobza et al.^62−65^

In contrast to the natural base pairs, the non-natural Hachimoji (B:S and P:Z) base pairs are energetically less stable in their HG-type geometries than in their WC-type geometries. As mentioned above, for the HG-type B:S base pair, we observed a nonplanar titled configuration. The significantly weaker interaction in this HG geometry (−21.9 kcal mol^–1^) vs the WC geometry (−39.2 kcal mol^–1^) is consistent with elongated intermolecular H-bond contact distances. For the HG-type P:Z base pair, the weaker interaction energy in the HG geometry (−19.6 kcal mol^–1^) vs the WC-type geometry (−33.4 kcal mol^–1^) is again consistent with longer intermolecular H-bond contacts. Although one N–H···O contact is medium length (1.815 Å), the other N–H···O contact is long (2.050 Å), and the remaining weak C–H···O H-bond is quite long (2.260 Å). Also note that both of the N–H···O contacts deviate significantly from the linear arrangement of the three atoms that would be optimal for a strong H-bond. Nevertheless, as in the case of the WC geometries, we continue to see a correlation between the sum of the closest-contact H-bond distances and the interaction energy, at least for A:T and P:Z: 6.389 Å and −18.2 kcal mol^–1^ for A:T and 6.125 Å and −19.6 kcal mol^–1^ for P:Z. G:C^+^ deviates from this trend (6.164 Å and −48.3 kcal mol^–1^ due to the aforementioned ion–dipole interactions), and B:S has only two significant H-bond-like contacts.

#### SAPT Energy Component Analysis

**Figure 4:**
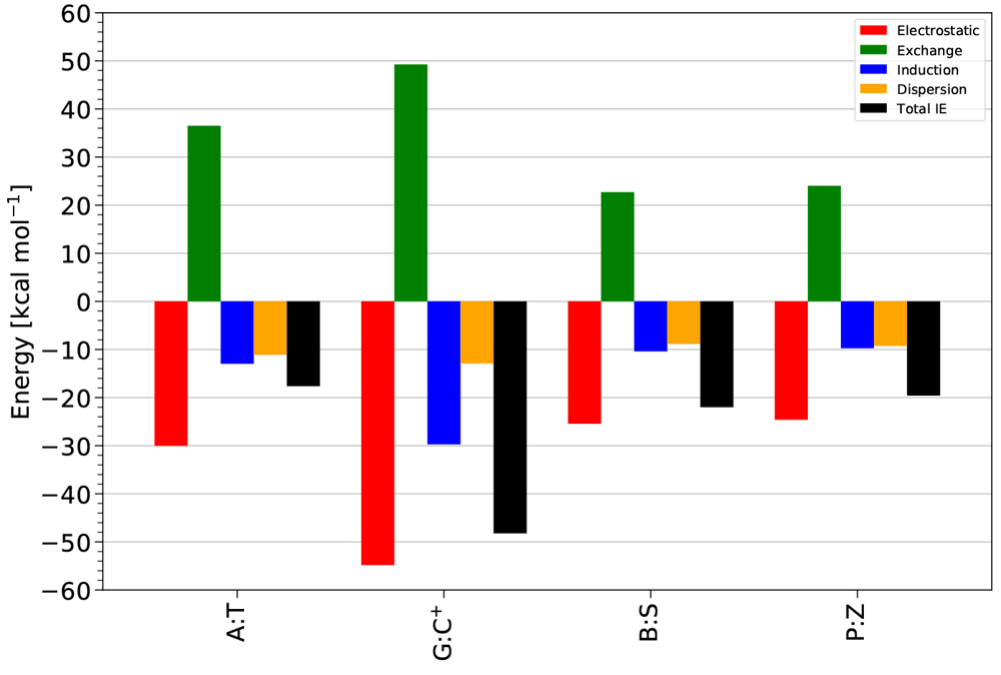
Total noncovalent interaction energy (total IE) values and SAPT components (electrostatic, exchange, induction, dispersion) for the HG (A:T, G:C^+^) and HG-type HM (B:S, P:Z) nucleic acid base pairs with the SAPT2+(3)(CCD)δMP2/aTZ basis set level of theory.

We analyzed the SAPT2+(3)(CCD)δMP2/aTZ energy components for the HG-type DNA base pairs (Figure 4). The largest energy component in Figure 4 is the electrostatic interaction between G and C^+^ (nearly twice as large as in B:S and P:Z). This is due to the very strong ion–neutral interaction in this base pair, as asserted above. We also note an unusually strong induction/polarization term for this base pair, as the ion–induced dipole contribution will be very large compared to dipole–induced dipole terms for the other base pairs. These strongly attractive contributions pull the bases close together (leading to two short H-bond contact distances noted above), thus also leading to an increase in the exchange-repulsion energy.

Looking more generally at Figure 4, it is interesting to note that the exchange-repulsion term is approximately the same size as the attractive electrostatics term (thus largely canceling it out), except for A:T, where it is slightly larger (by 6.5 kcal mol^–1^). For the WC geometries, exchange-repulsion was larger in magnitude than electrostatics for every base pair. This could be due to generally shorter H-bond distances in the WC geometries. Like in the WC base pairs, induction and dispersion components contribute significantly to the total interaction energies, with induction dominating over dispersion in all four base pairs, although the difference is <2.0 kcal mol^–1^, except for the G:C^+^ base pair where the difference is 16.8 kcal mol^–1^.

## Conclusions

Noncovalent forces play a critical role in determining biomolecular structure, including the structures of DNA and RNA. Here, we have presented a detailed, high-level theoretical examination of H-bonding in both natural (A:T and G:C) and non-natural Hachimoji (B:S and P:Z) DNA base pairs, in their standard Watson–Crick (WC) type geometries, and also in Hoogsteen (HG) type geometries. Interestingly, the non-natural Hachomoji-type base pairs interact more strongly than the corresponding natural base pairs, by −21.8 (B:S) and −0.3 kcal mol^–1^ (P:Z) in the WC geometries, according to coupled-cluster theory [CCSD(T)] in the complete-basis-set limit. The stronger interaction in B:S vs A:T is due to the formation of a third H-bond in B:S.

In addition, the natural base pairs are energetically more stabilized in their HG-type geometries than in their WC-type geometries: A:T by a minor −0.8 and G:C^+^ by −15.3 kcal mol^–1^. This stronger interaction of A:T in the HG-type geometry more than the WC-type geometry is consistent with the experimental infrared-spectra analysis of A:T dimers in a nonpolar solvent.^59−61^ By contrast, HG-type geometries are substantially *less* stable than WC-type geometries for non-natural Hachimoji DNA base pairs, B:S by 17.3 and P:Z by 13.8 kcal mol^–1^. We also noted a general correlation between the total interaction energy and the sum of the closest-contact H-bond distances (including the weak C–H···O contact in A:T and in the HG geometry of P:Z).

We have applied high-order quantum mechanical energy component analysis, using symmetry-adapted perturbation theory (SAPT), to understand the base pair interaction energies in terms of the physically meaningful components of electrostatics, exchange-repulsion, induction/polarization, and London dispersion. SAPT analysis confirmed that electrostatic interactions are the most attractive in base pair interactions, consistent with conventional wisdom about the nature of H-bonding interactions. However, the sum of induction and dispersion is nearly as large as electrostatics for both natural and non-natural base pairs, and for WC- and HG-type geometries. The magnitude of the SAPT components also tended to correlate with the sum of the closest-contact H-bond-type interactions. However, all SAPT terms are much larger in magnitude in the Hoogsteen G:C^+^ base pair than in any other base pair considered. This is consistent with very strong ion–dipole interactions in this complex which are absent in the other complexes, and it explains why the interaction energy is much stronger for this base pair even though it has only two true H-bonds.

The present interaction energies are expected to be the most accurate theoretical values reported to date for these important prototype systems, including for the newly proposed structures of Hoogsteen-type geometries for the non-natural Hachimoji base pairs. As such, the interaction energies may serve as valuable reference data. We also hope that the detailed energetic analysis of the interaction energy components, and how they relate to structure, may aid future studies of H-bonding between nucleobases and the design of additional non-natural nucleobases.

## Data Availability

The Supporting Information includes Cartesian coordinates for all geometries considered in this work. Most computations were performed using the open-source Psi4 quantum chemistry program, version 1.5, freely available from https://psicode.org. As noted in the Theoretical Methods section, geometry optimizations using B3LYP-D3(BJ) were peformed using the Q-Chem code, version 5.1, available from https://q-chem.com.

## Supporting Information Available

Scheme for torsion angles, MBIS atomic charge analysis, tables for comparison of aDZ/aTZ and CP/no-CP interaction energies, tables with interaction energies at different levels of SAPT and basis sets, SAPT interaction energy components, figures for the difference in total interaction energy values at some lower levels of SAPT for WC-, HM-, and HG-type natural and non-natural base pairs at different basis sets.(PDF)
Optimized geometries (xyz) for all base pairs at B3LYP-D3(BJ)/aDZ level of theory (ZIP)
