# Supplementary material for: High-Order Quantum-Mechanical Analysis of Hydrogen Bonding in Hachimoji and Natural DNA Base Pairs
Source: J Chem Inf Model. 2023 May 1;63(10):3150–7. doi: 10.1021/acs.jcim.3c00428 (PMC10207272; doi:10.1021/acs.jcim.3c00428)
Supplement: Supplementary file 1 — ci3c00428_si_001.pdf [file ci3c00428_si_001.pdf]

Supporting information for High-Order Quantum-Mechanical Analysis of Hydrogen Bonding in Hachimoji and Natural DNA Base Pairs

Rameshwar L. Kumawat and C. David Sherrill*

Center for Computational Molecular Science and Technology, School of Chemistry and Biochemistry, School of Computational Science and Engineering, Georgia Institute of Technology, Atlanta, GA 30332-0400

E-mail: sherrill@gatech.edu

In the following tables and figures the basis sets are abbreviated as follows: aug-cc-pVDZ = aDZ, aug-cc-pVTZ = aTZ.

Table S1: Comparison of the total interaction energy (in kcal mol^{-1}) of the WC-type natural (A:T, G:C) and non-natural HM (B:S, P:Z) base pairs computed at CCSD(T)/CBS[aQ5Z;δ:X], CCSD(T)/CBS[aTQZ;δ:X], and SAPT2+(3)(CCD)δMP2/X level of theory, where X = aDZ and aTZ. CP represents the use of the counterpoise correction.

Base Pairs	A:T		G:C		B:S		P:Z	
Methods	aDZ	aTZ	aDZ	aTZ	aDZ	aTZ	aDZ	aTZ
CCSD(T)/CBS[aQ5Z;δ:X](CP)	-17.17	-17.41	-32.70	-33.03	-38.83	-39.19	-33.02	-33.36
CCSD(T)/CBS[aQ5Z;δ:X](noCP)	-17.29	-17.33	-32.91	-32.96	-39.04	-39.10	-33.23	-32.26
CCSD(T)/CBS[aTQZ;δ:X](CP)	-17.16	-17.40	-32.68	-33.01	-38.81	-39.17	-33.00	-33.34
CCSD(T)/CBS[aTQZ;δ:X](noCP)	-17.46	-17.49	-33.11	-33.16	-39.29	-39.36	-33.49	-33.52
SAPT2+(3)(CCD)δMP2/X	-15.82	-16.87	-31.22	-32.82	-37.61	-39.30	-31.47	-33.11
CCSD(T)/CBS[aTQZ;δ:DZ][a]	-16.9	–	-32.1	–	–	–	–	–
MP2/X[a]	-14.8	-16.0	-28.7	-30.4	–	–	–	–
DFT-SAPT/X[b]	-14.1	-15.2	-28.1	-29.8	–	–	–	–

[a] Taken from Jurecka et al.[1] The authors used modified 6-31G** and cc-pVDZ basis sets for the △CCSD(T) correction, and they do not specify which one is used for A:T and G:C computations. [b] Taken from Andreas et al.[2]

Table S2: Comparison of the total interaction energy (in kcal mol^{-1}) of the HG-type natural (A:T, G:C^+) and HM non-natural (B:S, P:Z) base pairs computed at CCSD(T)/CBS[aQ5Z;δ:X], CCSD(T)/CBS[aTQZ;δ:X], and SAPT2+(3)(CCD)δMP2/X level of theory, where X = aDZ and aTZ. CP represents the use of the counterpoise correction.

Base Pairs	A:T		G:C^+		B:S		P:Z	
Methods	aDZ	aTZ	aDZ	aTZ	aDZ	aTZ	aDZ	aTZ
CCSD(T)/CBS[aQ5Z;δ:X](CP)	-17.97	-18.21	-48.03	-48.34	-21.68	-21.86	-19.39	-19.56
CCSD(T)/CBS[aQ5Z;δ:X](noCP)	-18.07	-18.09	-48.14	-48.20	-21.78	-21.76	-19.51	-19.45
CCSD(T)/CBS[aTQZ;δ:X](CP)	-17.96	-18.20	-48.01	-48.32	-21.67	-21.85	-19.38	-19.55
CCSD(T)/CBS[aTQZ;δ:X](noCP)	-18.28	-18.29	-48.43	-48.49	-21.93	-21.91	-19.76	-19.69
SAPT2+(3)(CCD)δMP2/X	-16.62	-17.63	-46.75	-48.22	-21.25	-22.00	-18.78	-19.59

Table S3: Comparison of the total interaction energy (in kcal mol^{-1}) of the WC-type natural (A:T, G:C) and HM non-natural (B:S, P:Z) base pairs at different levels of SAPT and basis sets.

Base Pairs	A:T		G:C		B:S		P:Z	
Methods	aDZ	aTZ	aDZ	aTZ	aDZ	aTZ	aDZ	aTZ
HF	-10.16	-10.29	-25.46	-25.71	-30.43	-30.69	-35.53	-25.84
SAPT0	-19.94	-20.89	-38.13	-39.53	-44.40	-45.97	-38.42	-39.88
SAPT2	-15.92	-17.43	-30.44	-32.61	-36.66	-39.06	-30.85	-33.04
SAPT2+	-17.35	-18.89	-32.40	-34.64	-38.82	-41.29	-31.77	-35.03
SAPT2+(3)	-16.90	-18.27	-32.55	-34.59	-38.70	-40.92	-32.89	-34.96
SAPT2+δMP2	-16.70	-17.97	-31.59	-33.45	-38.32	-40.34	-31.85	-33.75
SAPT2+(3)δMP2	-16.25	-17.35	-31.74	-33.41	-38.19	-39.97	-31.97	-33.68
SAPT2+(CCD)	-16.93	-18.41	-31.89	-34.06	-38.23	-40.62	-32.27	-34.46
SAPT2+(3)(CCD)	-16.47	-17.79	-32.04	-34.01	-38.11	-40.25	-32.39	-34.39
SAPT2+(CCD)δMP2	-16.27	-17.49	-31.07	-32.87	-37.73	-39.67	-31.35	-33.17
SAPT2+(3)(CCD)δMP2	-15.82	-16.87	-31.22	-32.82	-37.61	-39.30	-31.47	-33.11

Table S4: Comparison of the total interaction energy (in kcal mol^{-1}) of the HG-type natural (A:T, G:C^{+}) and HM non-natural (B:S, P:Z) base pairs at different levels of SAPT and basis sets.

Base Pairs	A:T		G:C^{+}		B:S		P:Z	
Methods	aDZ	aTZ	aDZ	aTZ	aDZ	aTZ	aDZ	aTZ
HF	-10.83	-10.96	-40.57	-40.88	-16.54	-16.65	-15.15	-15.19
SAPT0	-20.72	-21.65	-52.25	-53.54	-24.39	-25.09	-23.21	-23.91
SAPT2	-17.02	-18.48	-46.43	-48.29	-20.79	-21.84	-17.94	-19.03
SAPT2+	-18.35	-19.85	-47.72	-49.61	-21.90	-22.97	-19.14	-20.27
SAPT2+(3)	-17.76	-19.08	-48.04	-49.79	-21.67	-22.66	-19.44	-20.50
SAPT2+δMP2	-17.66	-18.89	-46.90	-48.56	-21.84	-22.72	-18.83	-19.76
SAPT2+(3)δMP2	-17.06	-18.12	-47.22	-48.74	-21.62	-22.41	-19.13	-19.99
SAPT2+(CCD)	-17.92	-19.36	-47.25	-49.08	-21.53	-22.57	-18.78	-19.87
SAPT2+(3)(CCD)	-17.32	-18.59	-47.57	-49.27	-21.31	-22.26	-19.09	-20.10
SAPT2+(CCD)δMP2	-17.22	-18.39	-46.43	-48.03	-21.47	-22.31	-18.47	-19.36
SAPT2+(3)(CCD)δMP2	-16.62	-17.63	-46.75	-48.22	-21.25	-22.00	-18.78	-19.59

Table S5: Total non-covalent interaction energy (Total IE, kcal mol^{-1}) and SAPT energy components for the natural WC- and HM-type nucleic acid base pairs at the SAPT2+(3)(CCD)δMP2/aTZ basis set level of theory.

Base Pairs	Electrostatic	Exchange	Induction	Dispersion	Total IE
A:T	-29.35	36.72	-13.10	-11.14	-16.87
G:C	-46.82	51.17	-22.49	-14.68	-32.82
B:S	-54.65	59.63	-28.11	-16.17	-39.31
P:Z	-46.69	51.96	-23.51	-14.87	-33.11

Table S6: Total non-covalent interaction energy (Total IE, kcal mol^{-1}) and SAPT energy components for the non-natural HG-type nucleic acid base pairs at the SAPT2+(3)(CCD)δMP2/aTZ basis set level of theory..

Base Pairs	Electrostatic	Exchange	Induction	Dispersion	Total IE
A:T	-30.03	36.50	-12.97	-11.13	-17.63
G:C^{+}	-54.83	49.26	-29.73	-12.92	-48.22
B:S	-25.44	22.70	-10.40	-8.86	-22.01
P:Z	-24.61	24.03	-9.75	-9.26	-19.59

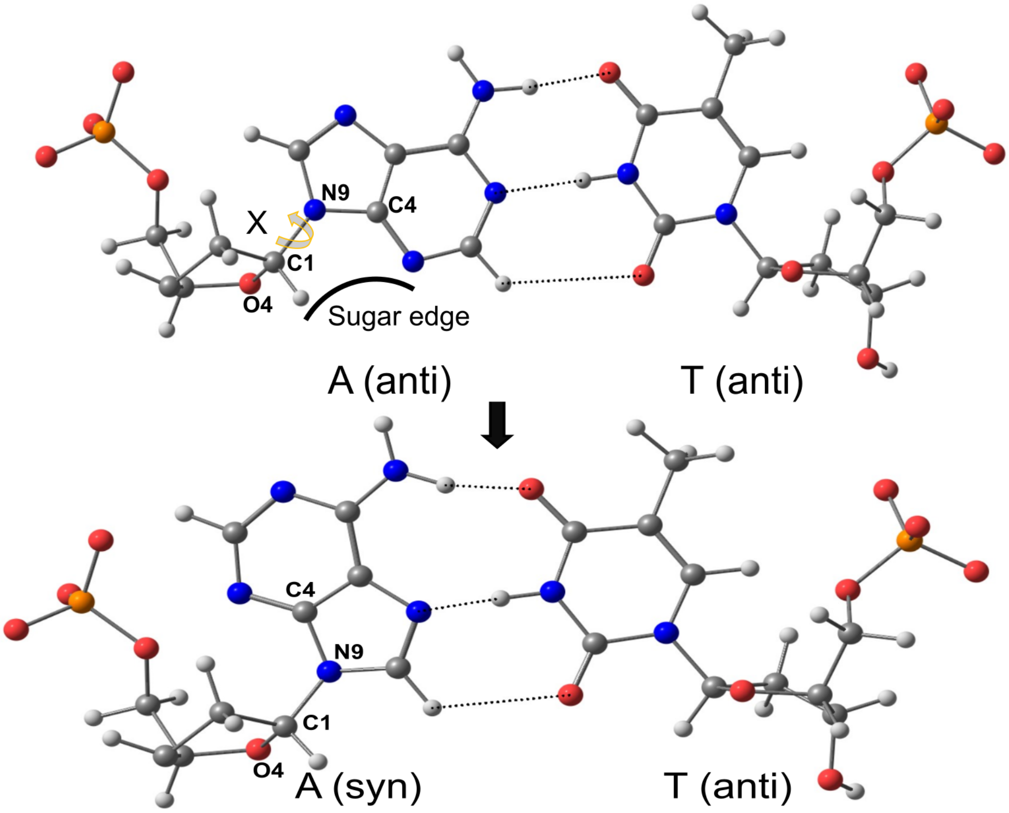

Figure S1: Schematic chemical structures representation of the chi (χ) torsion angle, which characterizes the relative base/sugar orientation, is defined by O4-C1-N9-C4 for purines (A and T).

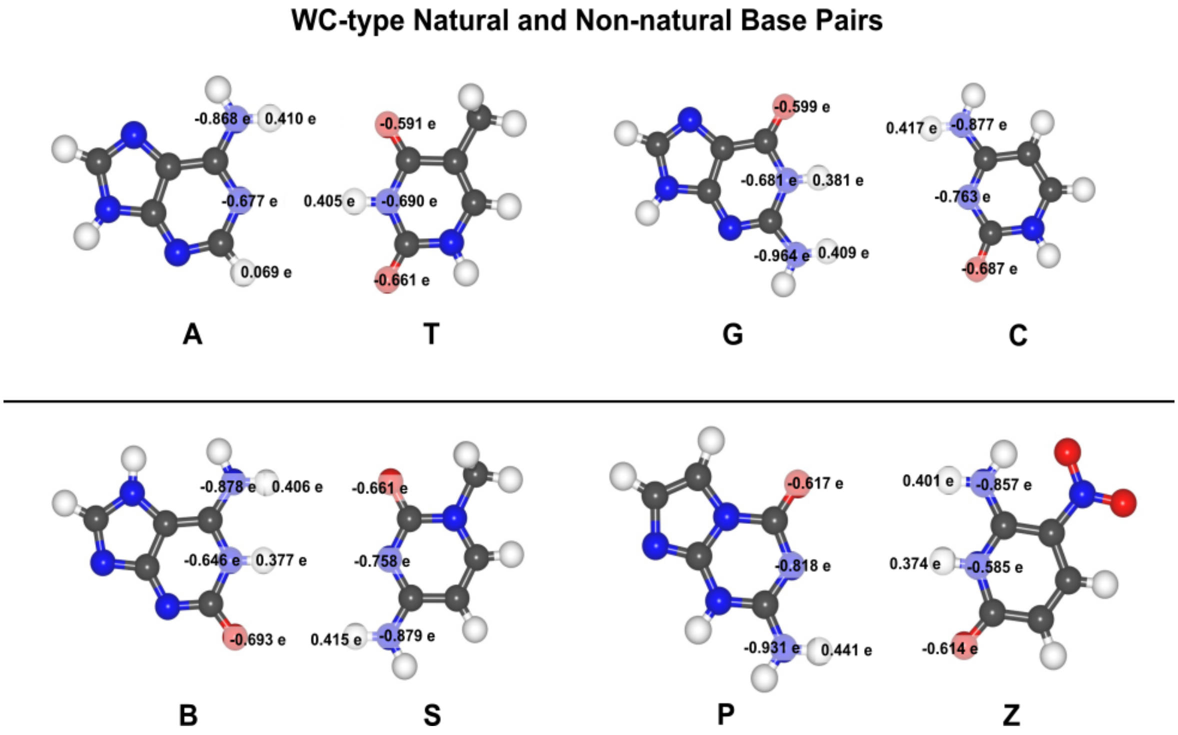

Figure S2: MBIS atomic charges for WC-type natural (A, T, G, C) and non-natural Hachimoji (B, S, P, Z) base pairs computed at B3LYP-D3(BJ)/aDZ basis set level of theory.

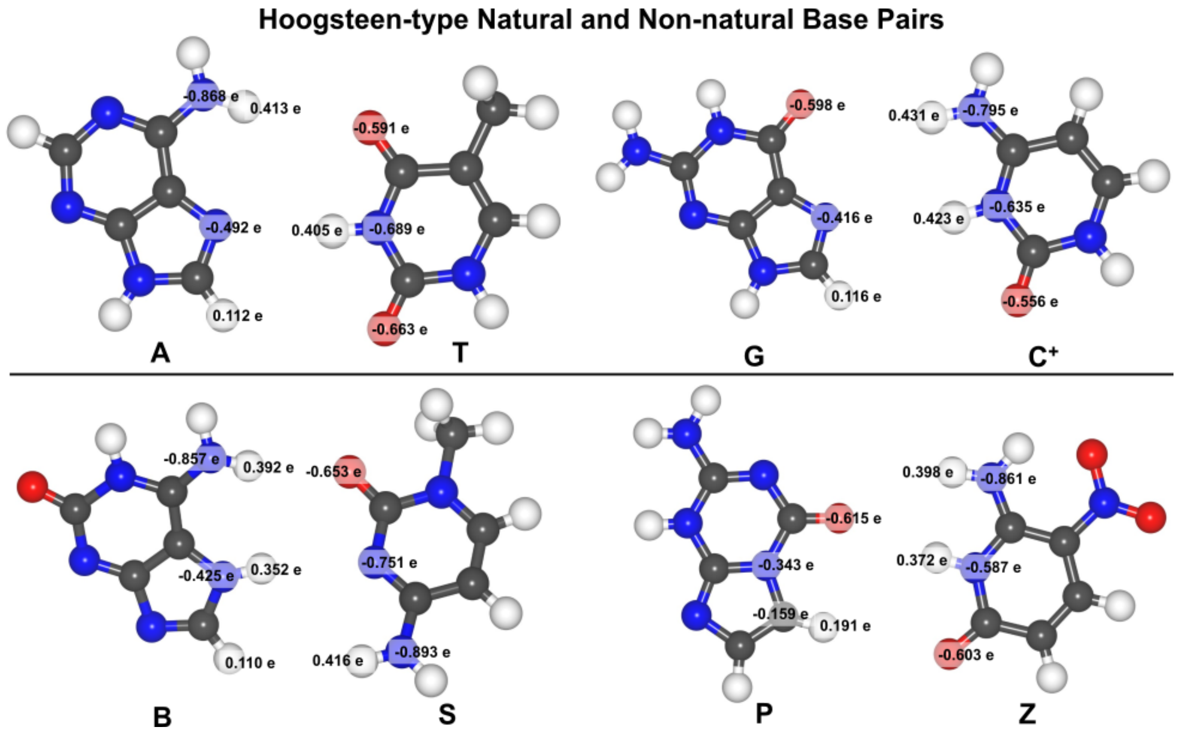

Figure S3: MBIS atomic charges for Hoogsteen-type natural (A, T, G, C^+) and non-natural Hachimoji (B, S, P, Z) base pairs computed at B3LYP-D3(BJ)/aDZ basis set level of theory.

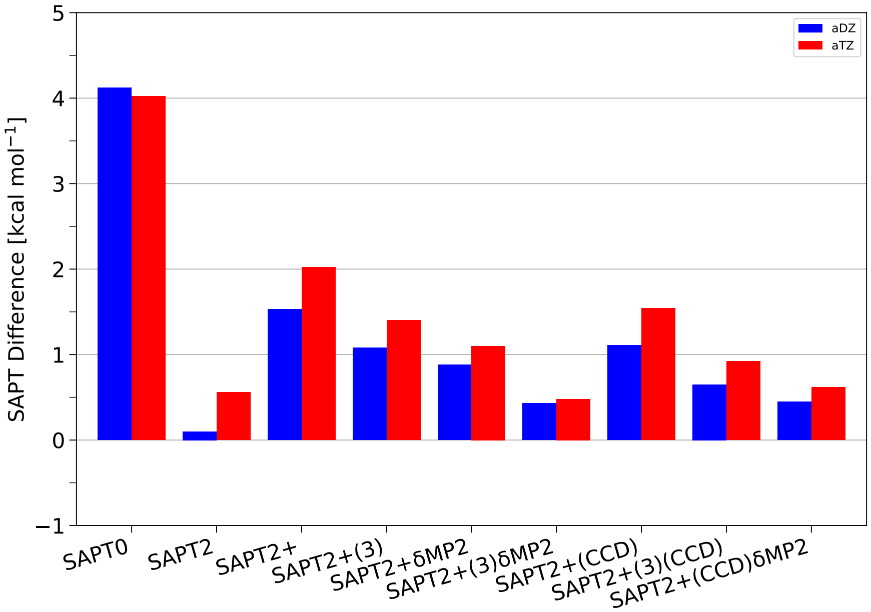

Figure S4: Difference in total interaction energy values at some lower levels of SAPT of WC-type natural A:T base pair with respect to high-order SAPT2+(3)(CCD)δMP2/aXZ, where X = D, T basis sets.

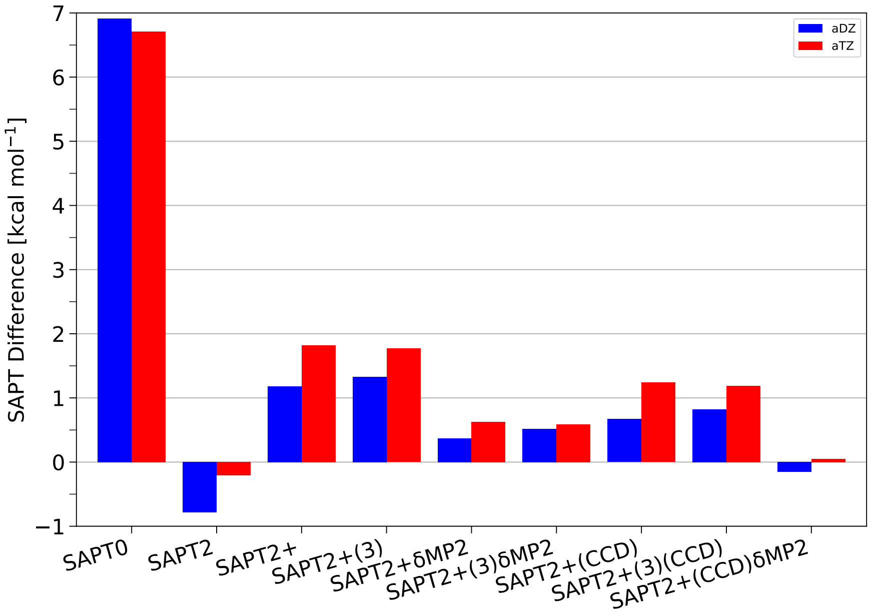

Figure S5: Difference in total interaction energy values at some lower levels of SAPT of WC-type natural G:C base pair with respect to high-order SAPT2+(3)(CCD)δMP2/aXZ, where X = D, T basis sets.

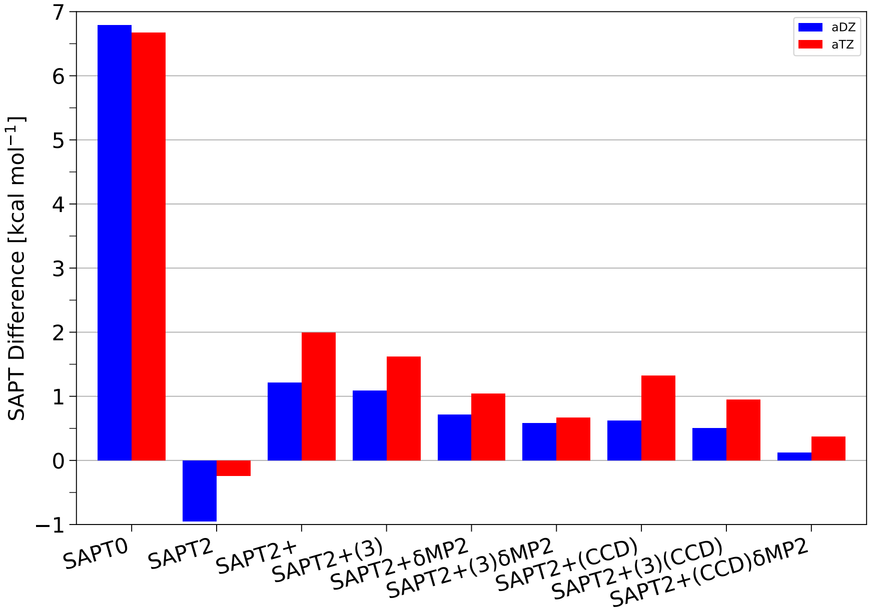

Figure S6: Difference in total interaction energy values at some lower levels of SAPT of WC-type HM non-natural B:S base pair with respect to high-order SAPT2+(3)(CCD)δMP2/aXZ, where X = D, T basis sets

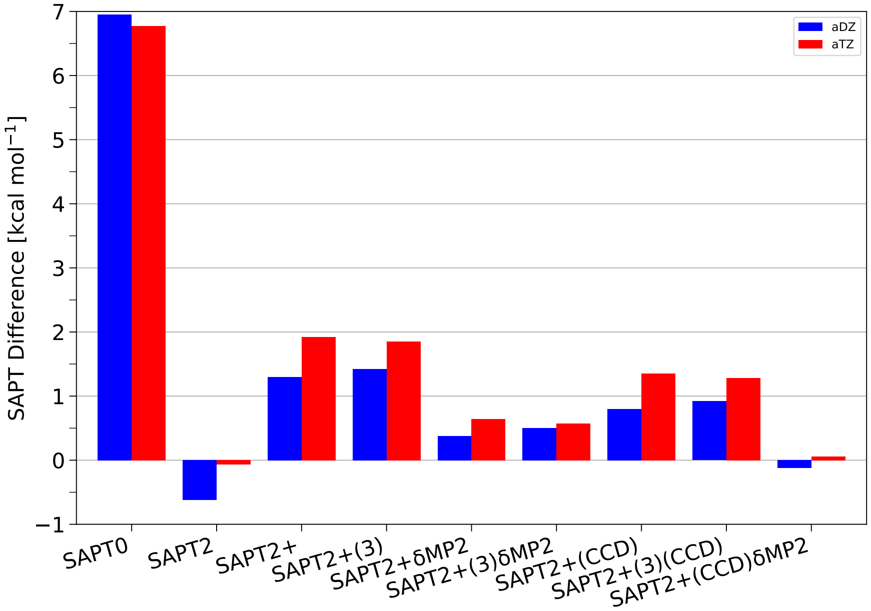

Figure S7: Difference in total interaction energy values at some lower levels of SAPT of WC-type HM non-natural P:Z base pair with respect to high-order SAPT2+(3)(CCD)δMP2/aXZ, where X = D, T basis sets.

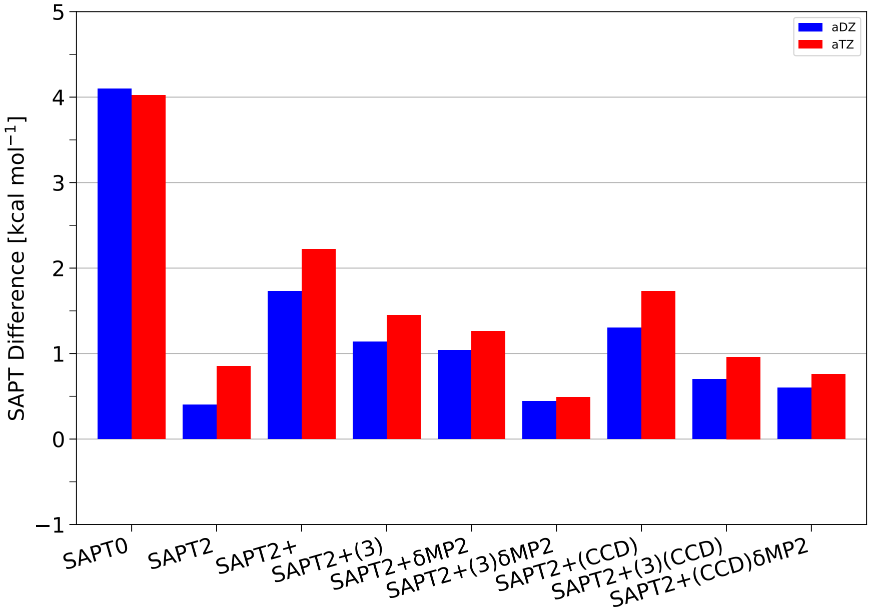

Figure S8: Difference in total interaction energy values at some lower levels of SAPT of HG-type natural A:T base pair with respect to high-order SAPT2+(3)(CCD)δMP2/aXZ, where X = D, T basis sets.

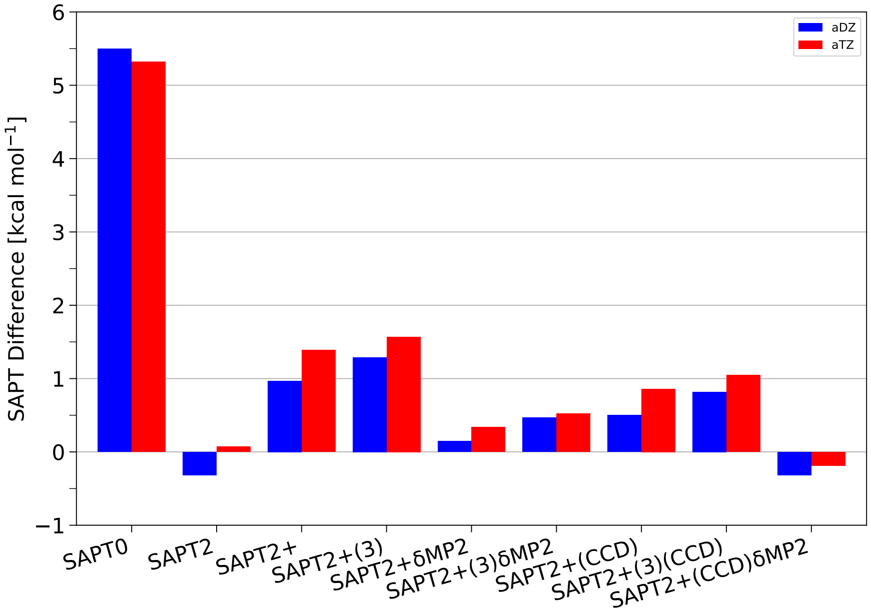

Figure S9: Difference in total interaction energy values at some lower levels of SAPT of HG-type natural G:C$^+$ base pair with respect to high-order SAPT2+(3)(CCD)δMP2/aXZ, where X = D, T basis sets.

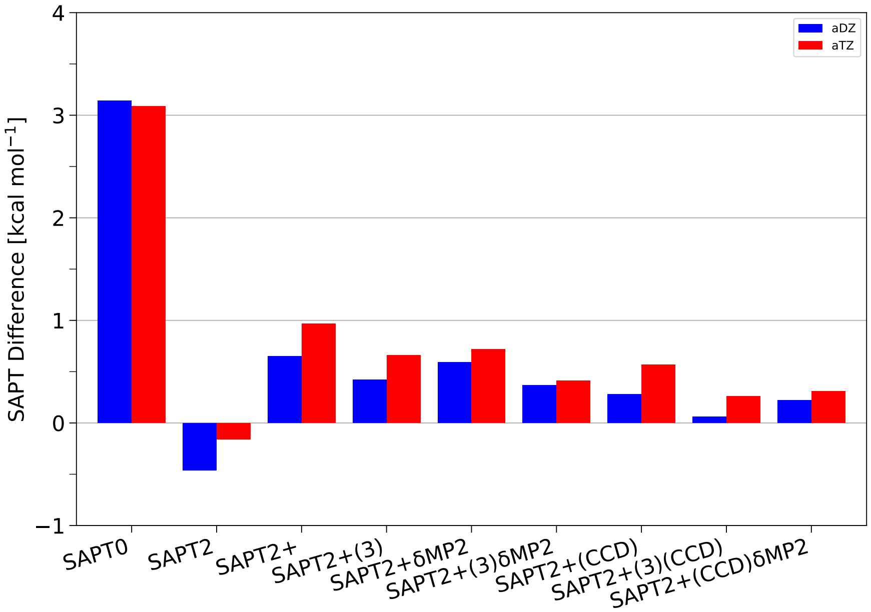

Figure S10: Difference in total interaction energy values at some lower levels of SAPT of HG-type HM non-natural B:S base pair with respect to high-order SAPT2+(3)(CCD)δMP2/aXZ, where X = D, T basis sets.

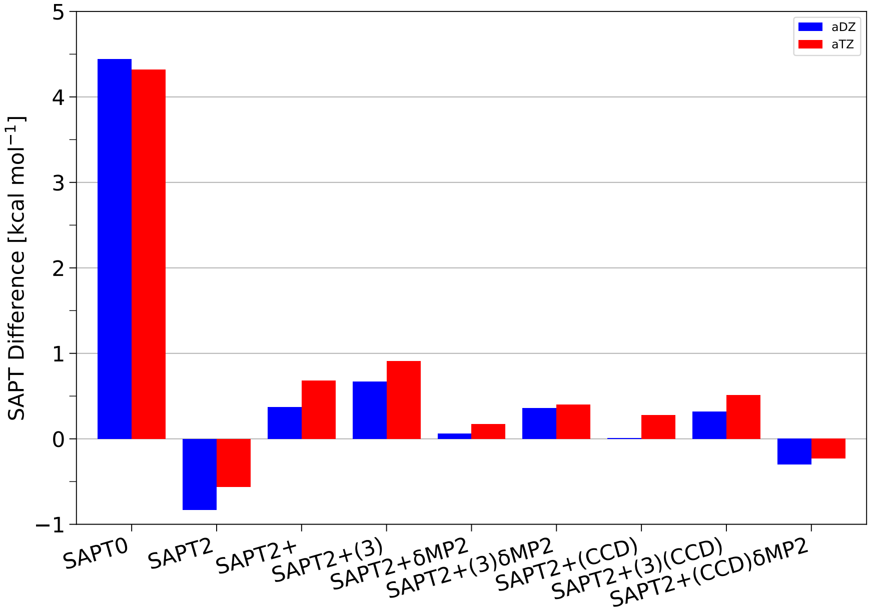

Figure S11: Difference in total interaction energy values at some lower levels of SAPT of HG-type HM non-natural P:Z base pair with respect to high-order SAPT2+(3)(CCD)δMP2/aXZ, where X = D, T basis sets.

References

(1) Jurečka, P.; Šponer, J.; Černỳ, J.; Hobza, P. Benchmark database of accurate (MP2 and CCSD (T) complete basis set limit) interaction energies of small model complexes, DNA base pairs, and amino acid pairs. *Phys. Chem. Chem. Phys.* **2006**, *8*, 1985–1993.

(2) Hesselmann, A.; Jansen, G.; Schütz, M. Interaction energy contributions of H-bonded and stacked structures of the AT and GC DNA base pairs from the combined density functional theory and intermolecular perturbation theory approach. *J. Am. Chem. Soc.* **2006**, *128*, 11730–11731.
